# Supraspinal neuroinflammation and anxio-depressive-like behaviors in young- and older- adult mice with osteoarthritis pain: the effect of morphine

**DOI:** 10.1007/s00213-023-06436-1

**Published:** 2023-08-02

**Authors:** Giada Amodeo, Silvia Franchi, Simona D’Agnelli, Giulia Galimberti, Marco Baciarello, Elena Giovanna Bignami, Paola Sacerdote

**Affiliations:** 1 Dipartimento Di Scienze Farmacologiche E Biomolecolari, University of Milan, Via Vanvitelli 32, 20129 Milano, Italy; 2 Anesthesiology, Critical Care and Pain Medicine Division, Department of Medicine and Surgery, University of Parma, Via Gramsci 14, 43126 Parma, Italy

**Keywords:** Chronic pain, Neuroinflammation, Mood alterations, Morphine, Aging, Monoiodo-acetate OA model

## Abstract

**Rationale:**

Asteoarthritis (OA) is a leading cause of chronic pain in the elderly population and is often associated with emotional comorbidities such as anxiety and depression. Despite age is a risk factor for both OA and mood disorders, preclinical studies are mainly conducted in young adult animals.

**Objectives:**

Here, using young adult (11-week-old) and older adult (20-month-old) mice, we evaluate in a monosodium-iodoacetate-(MIA)-induced OA model the development of anxio-depressive-like behaviors and whether brain neuroinflammation may underlie the observed changes. We also test whether an effective pain treatment may prevent behavioral and biochemical alterations.

**Methods:**

Mechanical allodynia was monitored throughout the experimental protocol, while at the end of protocol (14 days), anxio-depressive-like behaviors and cognitive dysfunction were assessed. Neuroinflammatory condition was evaluated in prefrontal cortex, hippocampus and hypothalamus. Serum IFNγ levels were also measured. Moreover, we test the efficacy of a 1-week treatment with morphine (2.5 mg/kg) on pain, mood alterations and neuroinflammation.

**Results:**

We observed that young adult and older adult controls (CTRs) mice had comparable allodynic thresholds and developed similar allodynia after MIA injection. Older adult CTRs were characterized by altered behavior in the tests used to assess the presence of depression and cognitive impairment and by elevated neuroinflammatory markers in brain areas compared to younger ones. The presence of pain induced depressive-like behavior and neuroinflammation in adult young mice, anxiety-like behavior in both age groups and worsened neuroinflammation in older adult mice. Morphine treatment counteracted pain, anxio-depressive behaviors and neuroinflammatory activation in both young adult and older adult mice.

**Conclusions:**

Here, we demonstrated that the presence of chronic pain in young adult mice induces mood alterations and supraspinal biochemical changes and aggravates the alterations already evident in older adult animals. A treatment with morphine, counteracting the pain, prevents the development of anxio-depressive disorders and reduces neuroinflammation.

**Supplementary Information:**

The online version contains supplementary material available at 10.1007/s00213-023-06436-1.

## Introduction

The world population over the age of 65 is estimated to significantly increase in the next 20 years. This growing population, therefore, shows the relevance of focusing research on the mechanisms and treatment of mental comorbidities in aging. Chronic pain and mood alterations, particularly depression, are two prevalent conditions among the elderly population (Zis et al. 2017). Osteoarthritis (OA) is a major musculoskeletal disorder affecting at least 50% of the elderly population, and pain is the main symptom, also occurring more commonly than stiffness or disability (Johnson and Hunter 2014). The increasing prevalence of OA is causing a dramatic increase in the number of people afflicted by chronic pain (Blackburn Cross et al. 2014)*.* In addition, as a consequence of pain and suffering, patients also experience related cognitive and affective disorders, including memory dysfunction, depression and anxiety. Emotional comorbidities further potentiate hypersensitivity with more rapid cartilage degradation and disability, thus fueling a vicious cycle that contributes to a worse quality of life in patients (Blackburn-Munro and Blackburn-Munro 2001). Different studies suggested that the underlying mechanisms involved in chronic pain processes are also implicated in memory deficits and psychiatric disorders due to a neurobiological overlap between pain processing and stressful and emotional signals (Zhuo 2016). In the last few years, these aspects started to be increasingly investigated in preclinical models of chronic pain. In animals with neuropathic pain, the presence of anxio-depressive-like behaviors was constantly reported (Amodeo et al. 2021b; Yalcin et al. 2011). Neuroinflammation and immune mechanisms are included among the factors implicated in pain pathophysiology (Malcangio 2019; Ní Chasaide and Lynch 2020; Guida et al. 2022). Indeed, in brain, the increased levels of proinflammatory molecules released by microglia and other glial cells, like astrocytes, can participate in maladaptive neural reorganization and contribute to both sensory and affective components of neuropathic pain (Barcelon et al. 2019; Guida et al. 2022). Less investigated, in animal models, is the relationship between OA pain and mood disorders, although few recent studies suggest that in monosodium-iodoacetate (MIA)-induced OA model, animals exhibit psychiatric-like features (La Porta et al. 2015; Carcolé et al. 2019; Batallé et al. 2021). However, these studies have been conducted only in relatively young adult animals, even if aging is the most relevant risk factor for OA development (O'Brien and McDougall 2019). For these reasons in the present study, we want to understand whether persistent OA pain in older adult mice may be related to cognitive and emotional disorders, compared with younger adults. We use the MIA-induced OA model, one of the most used in rodents and validated to assess pain and its treatments (Ogbonna et al. 2015; de Sousa Valente 2019; Amodeo et al. 2021a; 2022). MIA injection into the intra-articular space (IA) results in functional impairment that resembles human OA (de Sousa Valente 2019). MIA animals develop pain-related behaviors such as thermal hyperalgesia and mechanical allodynia (de Sousa Valente 2019; Amodeo et al. 2021a; 2022). To examine the pathways involved in the development of mood disorders, we analyzed in the prefrontal cortex (PFC), hippocampus (HPC) and hypothalamus (HPT) the levels of proinflammatory cytokines TNFα and IL-6, as well as the mRNA expression levels of TLR4, iba1 (microglia), GFAP (astrocytes) and the brain-derived neurotrophic factor (BDNF). These areas are key sites in mood control (Guida et al. 2022) and are important regulators of the affective and emotional components of pain (Khan et al. 2020). Indeed, neurochemical and morphological changes in these brain areas have been observed both in chronic pain conditions and in depressed subjects. The mediators and markers chosen are the most relevant and studied to define the presence of a neuroinflammatory state. In particular, glial activation is characterized by the upregulation of the cell-specific marker expression (such as Iba-1 for microglia and GFAP for astrocytes), of cell receptors involved in neuroinflammatory processes such as TLR4 as well as by the release of neuroactive cytokines such as TNFα, IL-6 and IL-10 (Bhattacharya and Jones 2018). Additionally, an increase in IL-6 and TNFα, and a decrease in BDNF have often been associated with anxiety and depression in patients as well as in experimental models (Pfau et al. 2018; Bhattacharya and Jones 2018; Barcelon et al. 2019; Amodeo et al. 2021b). Furthermore, it has recently been proposed that a supraspinal decrease in BDNF levels is associated with an increase in serum IFNγ, which could therefore be used as an early peripheral marker for depression (Chen et al. 2021). For this reason, the serum IFNγ levels were evaluated. A further important point that the study wants to address is whether prompt and efficacious pain treatment may modulate or prevent the development of psychiatric comorbidities in aging. In the elderly population, effective treatment of OA pain remains an important clinical concern (El-Tallawy et al. 2021). Opioids are potent analgesics and their efficacy also in OA pain is proven, but unwanted effects have limited their use (Pergolizzi et al. 2008; Ivers et al. 2012; Rastogi and Meek 2013; Friedman and Nabong 2020). In previous work with the same MIA model in young adult and older adult mice we demonstrated that a precocious treatment with morphine was able to relieve hypersensitivity and motor dysfunctions, and to counteract neuroinflammation in the peripheral nervous system and in spinal cord in both age groups (Amodeo et al. 2022). In the present work, we therefore want to go ahead with the research investigating whether OA hypersensitivity is also associated with mood disorders and supraspinal neuroinflammation, also evaluating the effect of 1-week morphine administration to verify the final ratio between risk and benefit of opioid treatment in aged animals.

## Materials and Methods

### Animals

In all the experiments, young adult (11-week-old, *n* = 112) and older adult (20-month-old, *n* = 112) male mice of the strain C57BL/6 J (Charles River Laboratories, Calco, Italy) were used. Mice were housed either single (young adult, *n* = 32; older adult, *n* = 32) or multiple (young adult, *n* = 80; older adult, *n* = 80) per cage (type II—26 cm × 20 cm × 14 cm) with sawdust litter, nesting material and environmental enrichment. The facility environmental conditions were: 12-h dark/light cycle, temperature of 22 ± 1 °C and humidity of 55 ± 10%. Animals were fed with standard pellet and tap water ad libitum. The old mice arrived at our animal facility at the age of 6 months and were housed until they reached 20 months of age (old age) (Amodeo et al. 2022). During aging, mice were subjected to periodic veterinary checks to assess their health state. At the end of this period, no mouse was excluded from the study. Instead, young adult animals arrived at our facility two weeks before the experiments. Both young adult and older adult mice, one week before the beginning of the experimental protocol, were handled through exposure to a passive hand, tickling, and hand restraint for a few minutes per day. Moreover, animals of both ages were divided into two experimental cohorts: anxiety cohort and depression cohort (for each cohort: young, n = 56; older adult, *n* = 56). The ARRIVE guidelines were followed in all animal experiments. The Italian Ministry of Health's Animal Care and Use Committee authorized the use of animals in these experiments (Authorization 180/2020 to PS) in accordance with the International Association for the Study of Pain and European Community (E.C.L. 358/118/12/86) standards. According to the 3R principles, every attempt was made to lessen suffering and limit the number of animals employed. Correct strategies to minimize potential confounders were applied. All procedures were performed on the same experimental days in young adult and older adult mice. Researchers were blind to treatment conditions when performing both behavioral tests and statistical analysis. The protocol of the study is illustrated in Supplementary Fig. 1.

### Induction of osteoarthritis

At day 0, young adult and older adult mice of both cohorts were randomized (coin flipping method) in two groups: control mice (CTR, saline-treated; young adult, *n* = 28; older adult, *n* = 28) and osteoarthritis mice (OA, monoiodo-acetate (MIA)-treated; young adult, *n* = 28; older adult, *n* = 28) (Amodeo et al. 2021a, 2022). Under general anesthesia, mice were injected, by a single intra-articular (IA) administration into the right knee, with 1 mg of MIA (Sigma-Aldrich, Italy) in 10 μl of sterile saline (MIA-mice) or vehicle (CTR-mice) (Amodeo et al. 2022). MIA was injected by expert researchers, using great attention to avoid substance leakage from the articular capsule, which would cause systemic toxicity with consequent animal death.

### Morphine treatment

At day 7 of the experimental protocol, CTR- and MIA- mice of both ages and cohorts were randomized into morphine or saline groups (*each group, n* = *14)*. Morphine hydrochloride (Salars, Como, Italy), at the dose of 2.5 mg/kg, or saline (10 µl/g of mouse) were subcutaneously administered once daily for 7 days (from day 7 to day 13) (Amodeo et al. 2022). Animals received the last morphine injection the day before sacrifice.

### Behavioral testing

Before each behavioral test, mice were acclimated to the new room for 30 min. All behavioral evaluations were performed before the daily morphine injection. In all animals of both cohorts Von Frey test was performed at day 0, 3, 7, 10 and 14 of the experimental protocol. Depression- (sucrose preference test, novel object recognition test -NORT, tail suspension test -TST and forced swim test -FST) and anxiety- (open field test, light/ dark box test and marble burying test) like behavior tests were performed, in the respective cohort, at day 14. The mood-like behavior tests were performed from the least to the most impacting for the animal and between one test and the next, the animal was given an adequate amount of time for rest (a few hours). All depression- and anxiety-behavior tests were recorded and analyzed by ANY-maze video tracking software (v 7.1). The tests were performed in the light phase, always in the morning.

#### Pain-like behavior

*Von Frey test*: to assess mechanical allodynia, a dynamic plantar aesthesiometer (Ugo Basile, Italy) was used. Briefly, each animal was placed in a plexiglass test box (w 8.5 × h 8.5 cm) located on a metal mesh and left undisturbed for 15 min. To evaluate sensitivity to mechanical touch (punctate stimulus), the stiff and blunt tip of a von Frey filament (Ø 0.5 mm) was applied, with increasing force (ranging up to 10 g in 10 s), in the mid-plantar of the hind paws of the animals. The test ended at the cut-off (10 g) or when the animal, removed the paw. Both ipsilateral- (IA injection in the respective knee) and contralateral- (without IA injection in the respective knee) paws were tested (3 measurements for each paw). For every mouse, the mean of values obtained from the ipsilateral paw was calculated and used for statistical analysis. Paw withdrawal thresholds (PWT) were expressed in grams (g) (Amodeo et al. 2021a, 2021b, 2022).

#### Anxiety-like behavior

*Open field test:* the open field test is traditionally used to assess exploratory behavior, locomotion and anxiety-related behaviors. Indeed, increased anxiety leads to a decrease in exploratory behavior, which translates into less locomotor motion and preference for field edges rather than central zone. An apparatus (47,150-Ugo Basile, Italy), composed of 4 arenas (46 × 46 x h 40 cm) in gray plastic, was used. The mouse was placed in the middle of the arena (1 mouse per arena) and allowed to roam around freely for 10 min. The total distance traveled in the arena (meters) and the total time spent in the center zone (seconds) were used for statistical analysis (Moschetti et al. 2019).

*Light/ dark box test:* the light/ dark box test is founded on a natural conflict condition typically of rodents, i.e. their innate aversion to brightly illuminated spaces and the spontaneous exploratory behavior for a novel environment. Therefore, the conflict is the tendency to explore the new space despite being exposed to intense light. Briefly, the box test consisted of two plastic chambers communicating through a door (5 × 5 cm). One room was black and not exposed to light (l 12 × d 40 × h 50 cm, 1/3 of the box) while one room was white and exposed to lighting (l 24 × d 40 × h 50 cm, 2/3 of the box). The animal was located in the light chamber and allowed to freely travel between the two chambers for 5 min. The latency (i.e. the time spent for the first time in the white chamber before going into the dark chamber (seconds)), the total time spent in the light chamber (seconds) and the number of transitions between one chamber and another (number) were used for statistical analysis. Classically, a decrease in latency, time in the white arena and number of transitions reflects anxiety-like behavior (Amodeo et al. 2021b).

*Marble burying test*: through the marble burying test, both obsessive–compulsive behavior (intended as a high degree of repetitive behaviors, including digging) and anxiety (understood as a high degree of excavation, potentially as a means of attempting to escape the new environment) may be studied. Briefly, 5 cm of sawdust bedding was placed in new home cages and for each cage, on the surface 18 glass marbles were placed in a regular pattern, evenly spaced. The animal was put in the test cage (1 mouse for cage) and left undisturbed for 30 min. Following, the mouse was removed from the test cage and put back in its home cage. The number of marbles unburied (> 1/3 outside the sawdust) was counted (Amodeo et al. 2021b). For the statistical analysis, the percentage of non-buried marbles was used, calculating it according to the formula: non-buried marbles / (buried marbles + non-buried marbles). In animals in an anxious state, the % of non-buried marbles decreases.

#### Depression-like behavior

*Sucrose preference test*: the mice, individually housed in cage, were accustomed for 48 h to the presence of two bottles filled up with water (habituation period—from day 11 to day 13). Subsequently, at day 13, mice were exposed for 24 h to two bottles, one containing water and one containing 2% sucrose solution (test period). The bottles were placed at 8 AM, they were then switched into position to reduce any confusion produced by a lateral distortion around 8 PM, and the read-out of the intake was performed the next morning. Both bottles were weighed before and after the test period. For each animal, sucrose preference was calculated as a percentage of: volume of sucrose intake / (volume of sucrose intake + volume of water intake) (Moschetti et al. 2019; Amodeo et al. 2021b).

*Tail suspension test*: the tail suspension test is a rodent behavioral paradigm that measures behavioral despair or “depression-like” behavior and learned helplessness (Can et al. 2012). Briefly, three-walled rectangular gray plastic tail suspension boxes (h 55 × w 20 × d 12 cm) were used. A stainless-steel suspension bar (Ø 1 cm) was positioned on the top of the boxes. All animals of the depressive cohort were tested. Each mouse was attached, in the middle of the compartment, by the tail to the suspension bar (with adhesive tape). The distance between the mouse's nose and the floor was 20 cm. The test’s duration was of 6 min, during which the animal's behavior was observed, and resulting escape-oriented behaviors (mobility, in seconds) were used for statistical analysis.

*Forced swim test*: the forced swim test (known also as the Porsolt test) is based on the assumption that the animal, during aversive/stressful condition, will try to escape it. If escape is impossible, the animal normally stops trying and gives up. However, relatively quick and prolonged surrender is considered a manifestation of depressive condition. As test apparatus were used 3L glass beakers (h 27 cm, Ø 14.5 cm) filled with water (23 ± 2 °C). The animal was gently placed in the water and allowed to swim undisturbed for 6 min. Following this period, it was removed, wiped up and returned to its cage. Testing was conducted on all mice of the depressive cohort. Latency time, i.e. first time of prolonged immobility (> 10 s) and total immobility time during the last 4 min of the test, both expressed in seconds, were used for statistical analysis (Moschetti et al. 2019; Amodeo et al. 2021b).

*Novel object recognition test*: depression, although it is classically considered an "affective" disorder, includes both emotional and cognitive dysfunction, which may be independent of each other. In all animals of the depression cohort non-spatial learning was evaluated through the Novel Object Recognition (NOR) test. This test is based on the innate tendency of rodents to spend more time exploring a new object than a familiar one; therefore, the choice to explore the new object indicates learning memory and recognition, while a reduction in this preference indicates a cognitive disorder. The test arena consisted of a square plastic chamber (46 × 46 × 40 cm), with black walls and white flooring. The test was divided in 3 phases. T0, habituation session: at day 13, the mouse was placed in the center of the arena and left free to explore it for 5 min; T1, training session: at day 14, two identical objects were placed in the arena equidistant from the walls and from each other and then the mouse was put in the center of the arena and given 10 min to examine the two objects; T2, test session: after 60 min from the training session, a familiar object and a new object were positioned in the arena and the mouse was located in the center of the arena, free to examine the two objects for 10 min (Lueptow 2017). The discrimination index, expressed as the ratio between the difference in the time spent exploring the new object (Tn) compared to the old one (Tf) and the total amount of exploration time (DI = Tn–Tf/Tn + Tf) and the total exploration time (seconds) of the new object, during the test session (T2), was used for the statistical analysis.

#### Tissue collection

At the end of the experimental protocol, day 14 post IA injection, mice were killed by decapitation and blood and brains were obtained. Carotid artery blood was collected and incubated for 3 h at room temperature and subsequently for 2 h at 4 °C, in order to allow coagulation. Subsequently, blood was centrifuged at 21.000 g for 30 min at 4 °C. Serum was removed and stored at -80 °C. From the brain, important areas involved in pain and mood disorders, i.e. prefrontal cortex (PFC), hippocampus (HPC) and hypothalamus (HPT) were isolated, snap-frozen in liquid nitrogen and stored at − 80 °C.

### RT-qPCR

From the brain areas, RNA was extracted by homogenizing the tissue with the TRIzol® reagent, according to the manufacturer's instructions (Invitrogen, Carlsbad, USA). The cDNA was obtained using the LunaScript ™ Reverse Transcriptase Kit, according to the manufacturer's instructions (BioLabs, UK). mRNA levels of Interleukin 6 (IL-6) and Tumor Necrosis Factor Alpha (TNFα), Toll-like receptor 4 (TLR4), microglia (iba1), astrocytes (GFAP) and brain-derived neurotrophic factor (BDNF) were measured with quantitative RT-PCR by QuantStudio 5™ (Thermofisher Scientific, USA) using Taqman Gene expression assays ((Thermofisher Scientific, USA—IL-6: Mm00446190_m1, TNFα: Mm00443258_m1, TLR4: Mm00445274_m1, iba1: Mm00479862_g1, GFAP: Mm01253033_m1, BDNF: Mm04230607_s1) and Luna® Universal Probe qPCR Master Mix (BioLabs, UK), respecting the instruction of the manufacturers. The mRNA levels of the interest genes were normalized to GAPDH (Mm99999915_g1) and expressed as 2 − ΛΛCT relative to the control group (young adult CTRs). Each sample was run in triplicates alongside non-template controls.

### Total protein determination and ELISA assay

For protein extraction, brain areas were homogenized in ice-cold PBS supplemented by 0.05% EDTA and 0.5% protease inhibitor cocktail (Roche Diagnostics, Italy). The homogenates were centrifuged at 22.000 g for 15 min at 4 °C and the supernatants were collected. The IL-6, TNFα and IL-10 levels were assessed by ELISA assay (Invitrogen™, Thermofisher Scientific, USA), and the total protein content was measured with Lowry’s method (Amodeo et al. 2022). For each sample, the ratio of cytokine content (pg) to total protein content (mg) was calculated and reported as pg/mg. In serum samples, IFNγ levels were determined by ELISA according to the manufacturer's instructions (Invitrogen™, Thermofisher Scientific, USA). Data are reported as pg/ml.

### Statistical analysis

Statistical analysis was performed using GraphPad Prism 9 (San Diego, CA). Prior to statistical analysis, in each behavioral and biochemical test, normality was verified by the D'Agostino & Pearson test and the results did not indicate any violation of the assumption (regardless of single or multiple cage housing, or to animals from different cohorts). Additionally, to avoid experimental bias during the data analysis, for each parameter analyzed in each experimental group, a targeted statistical analysis (t-test) was first performed to exclude the presence of alterations due to the variables related to single or multiple cage housing and animals from different cohorts. Pain behavioral results (mechanical allodynia) were analyzed using Two-way repeated-measures ANOVA with Bonferroni’s post hoc test. Data represent mean ± SD of 28 animals/ group (i.e. 14 mice of anxiety cohort and 14 mice depressive cohort (6 multiple housing and 8 single housing)). For NORT, TST and FST the results were analyzed using One-way ANOVA with Bonferroni’s post hoc test. Data represent mean ± SEM of 14 animals/ group (6 mice housed in multiple cages and 8 mice housed in single cages). For the sucrose preference test the results were analyzed using One-way ANOVA with Bonferroni’s post hoc test. Data represent mean ± SEM of 8 animals/ group (all mice housed in single cages). For the anxiety-like behavior the results were analyzed using One-way ANOVA with Bonferroni’s post hoc test. Data represent mean ± SEM of 14 animals/ group (all mice housed in multiple cages). mRNA data were analyzed using One-way ANOVA followed by Bonferroni's test. Data represent mean ± SEM of 14 animals/group (7 mice of anxiety cohort and 7 mice of depression cohort (3 mice housed in multiple cages and 4 mice housed in single cages)). Protein data (brain areas) were analyzed using One-way ANOVA followed by Bonferroni's test. Data represent mean ± SEM of 8 animals/ group (4 mice of anxiety cohort and 4 mice of depression cohort (2 mice housed in multiple cages and 2 mice housed in single cages)). Serum data were analyzed using One-way ANOVA followed by Bonferroni's test. Data represent mean ± SEM of 22 animals/group (11 mice of anxiety cohort and 11 mice of depression cohort (5 mice housed in multiple cages and 6 mice housed in single cages). Pearson correlation test was used to verify the presence, strength and direction of the relationship between two different variables (cytokine protein content and BDNF levels in supraspinal areas (number of XY pairs = 64); IFNγ serum levels and BDNF in supraspinal areas (number of XY pairs = 112); IFNγ serum levels and immobility time in FST (number of XY pairs = 112)). For correlation analyses, all mice were considered, regardless of age or treatment. Pearson correlation coefficient, r, was reported. For all analyses, differences were considered significant at *p* ≤ 0.05.

## Results

### Behavioral evaluations

**Fig. 1:**
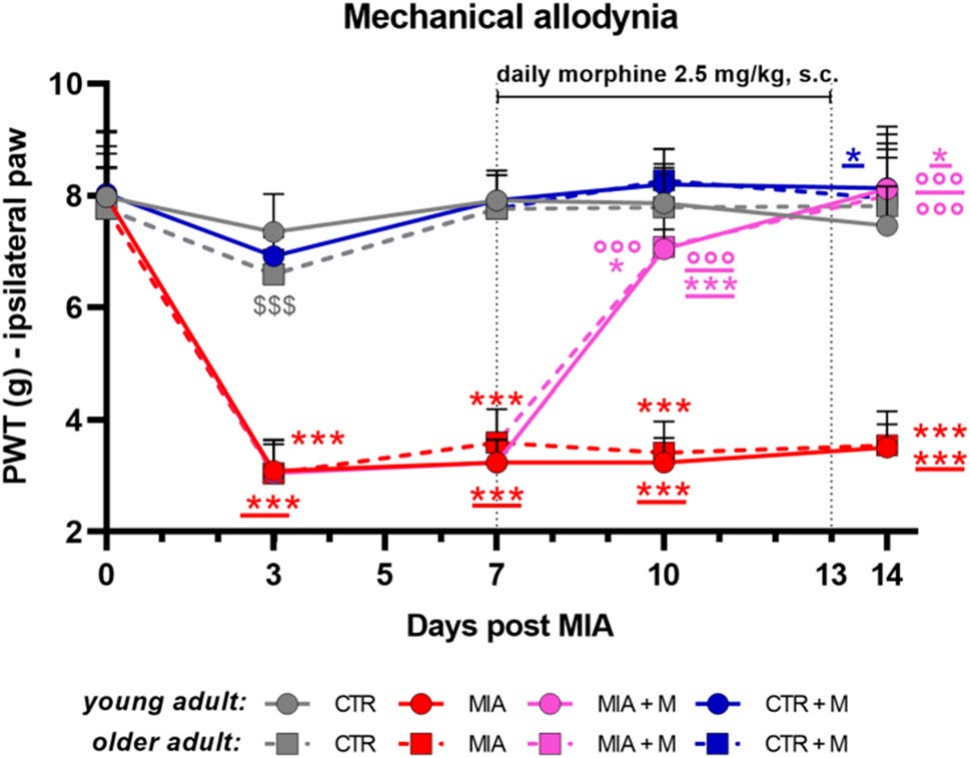
Time course of mechanical allodynia development. In young adult (11-week-old) and older adult (20-month-old) mice, OA was induced by single intra-articular administration of MIA (1 mg in the right knee, day 0). Morphine treatment (2.5 mg/kg, s.c.) was administered once daily for 1 week (from day 7 to day 13). The withdrawal thresholds of the ipsilateral paw to the touch of the Von Frey filament were monitored over time. Data represent mean ± SD of 28 mice/group. Statistical analysis was performed by means of Two-way ANOVA for repeated measures followed by Bonferroni's post-test. Time x Treatments: F (28, 864) = 95.53; *p* < 0.0001. *p < 0.05, ***p < 0.001 vs CTR older adult; *p < 0.05, ***p < 0.001 vs CTR young adult; °°°*p* < 0.001 vs MIA older adult; °°°*p* < 0.001 vs MIA young adult; $$$*p* < 0.001 vs respective young adult treatment group

As reported in Fig. 1, the basal PWT did not differ in young adult and older adult mice before MIA injection.

**Fig. 2:**
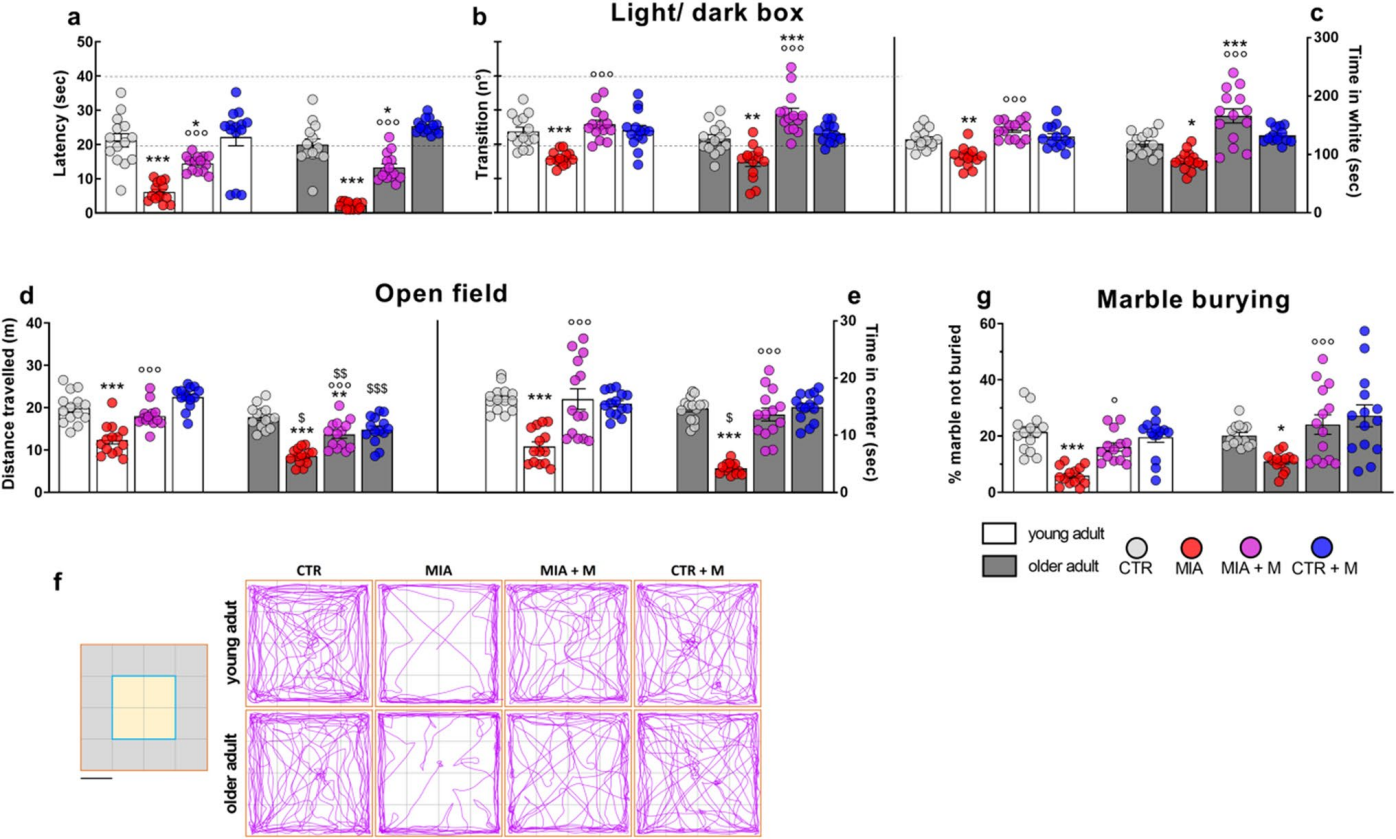
Anxiety-like behaviors. Behavioral evaluations were performed at the end of the experimental protocol, at day 14 (MIA IA administered at day 0, 1 mg in 10 µl in the right knee; morphine, s.c. administered, 2.5 mg/kg, once daily, from day 7 until day 13). The anxious state was assessed by means of light/dark box test, evaluating (**a**) the latency, i.e. the time before entering the dark chamber, (**b**) the number of transitions between the white and dark chamber and (**c**) the total time spent in the white chamber. By means of open field test were assessed (**d**) the total distance traveled and (**e**) the total time spent in the center of the arena. (**f**) Two areas were identified and used to interpret tracking data for thigmotaxic, the border area in gray and the central area in yellow. Scale bar: 11.5 cm. Representative traces of all experimental groups are shown. (**g**) By means of marble burying test was calculated the percentage of marbles not buried. Data are presented as mean ± SEM of 14 animals per group. Statistical analysis was performed by One-way ANOVA analysis of variance followed by Bonferroni’s post-test. Treatment: F (7, 104) = (a) 33.93, *p* < 0.0001; (**b**) 15.59, *p* < 0.0001; (c) 16.60, *p* < 0.0001; (**d**) 31.27, *p* < 0.0001; (**e**) 23.72, *p* < 0.0001; (**g**) 9.97, *p* < 0.0001. *p < 0.05, **p < 0.01, ***p < 0.001 vs respective age-CTR; °*p* < 0.05, °°°*p* < 0.001 vs respective age-MIA; $*p* < 0.05, $$*p* < 0.01, $$$*p* < 0.001 vs respective young adult treatment group

The intra-articular injection of MIA elicited significant mechanical allodynia (*p* < 0.001) in the ipsilateral paw of both young adult and older adult animals without significant differences related to age. No changes were present in the contralateral paw (data not shown). Seven days after MIA, when hypersensitivity was fully developed, animals were daily treated with 2.5 mg/kg of morphine for 7 days. One week of drug administration was able to fully reverse hypersensitivity in MIA mice, with no age difference (*p* < 0.001 vs MIA mice of respective age). Morphine did not affect PWT in healthy control animals. At the end of the experimental protocol, 14 days after MIA injection and 7 days of morphine chronic treatment, we assessed the presence of mood disorders. As reported in Fig. 2, in the anxiety cohort we performed light/ dark box (panels a-c), open field (panels d-f) and marble burying (panel g) tests. We did not observe differences between CTR young adult and older adult animals in any of the tests performed, indicating that aging does not affect the anxiety state of mice. However, the presence of OA induced significant anxiety-like behavior in young adult and older adult mice. This was reflected in all tests. In the light/ dark box test we observed, in both young adult and older adult MIA mice, a shorter latency to enter the dark chamber (panel a—CTR vs MIA young adult, *p* < 0.001; CTR vs MIA older adult, *p* < 0.001), a reduced number of transitions between the two chambers (panel b—CTR vs MIA young adult, *p* < 0.001; CTR vs MIA older adult, *p* < 0.01) and a significant reduction in the total time spent in the white chamber (panel c—CTR vs MIA young adult, *p* < 0.01; CTR vs MIA older adult, *p* < 0.05).

**Fig. 3:**
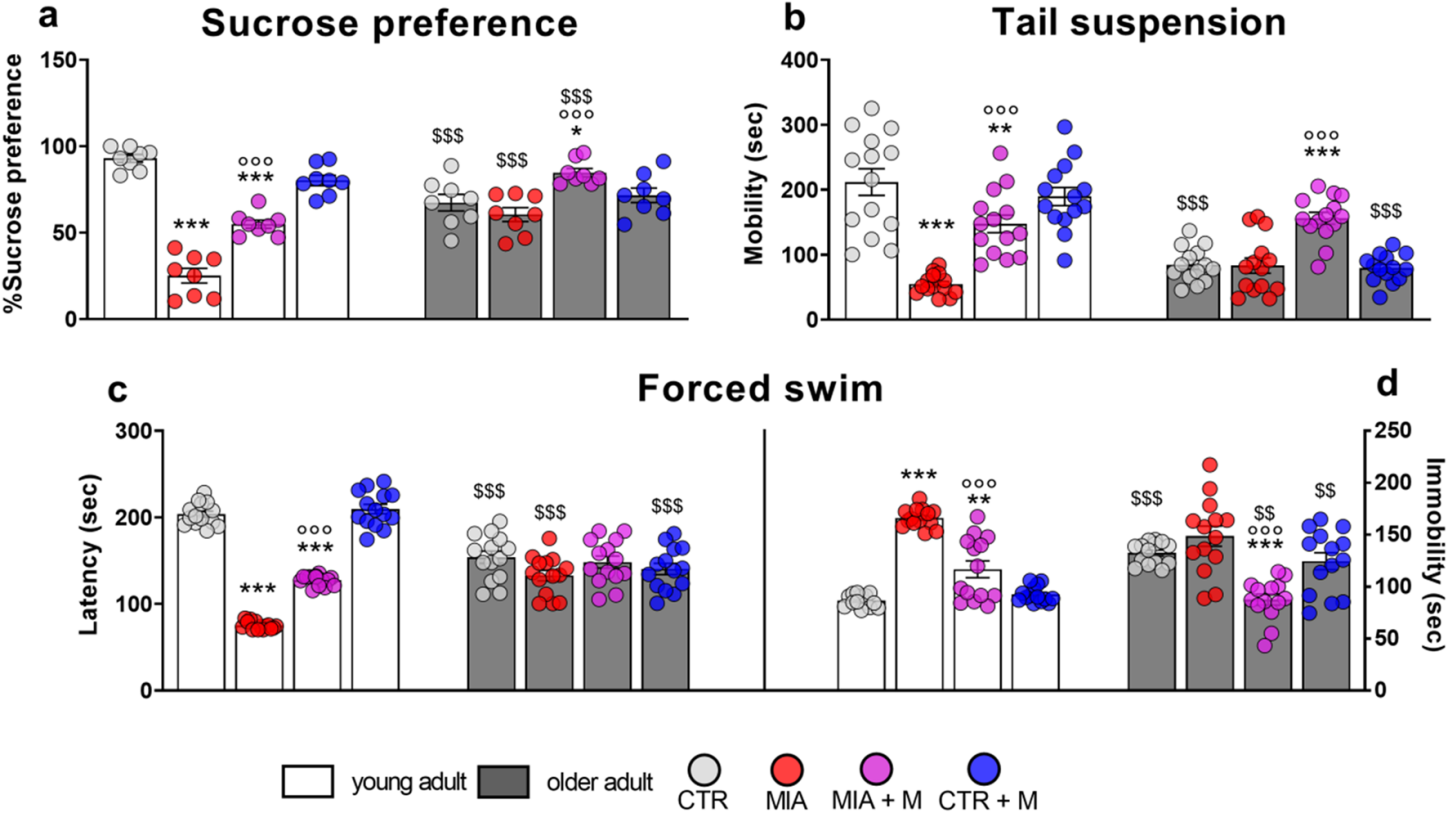
Depression-like behaviors. Behavioral evaluations were performed at the end of the experimental protocol, at day 14 (MIA IA administered at day 0, 1 mg in 10 µl in the right knee; morphine, s.c. administered, 2.5 mg/kg, once daily, from day 7 until day 13). The depressive state was assessed by means **(a)** of sucrose preference evaluation; by means of **(b)** tail suspension test assessing the total mobility time; and by means of forced swim test, evaluating **(c)** the latency, corresponding to the first stop time, and **(d)** the total immobility time in the last 4 min of the test. Data are presented as mean ± SEM of 8 (a) or 14 (b-d) animals per group. Statistical analysis was performed by One-way ANOVA analysis of variance followed by Bonferroni’s post-test. Treatment: **(a)** F (7, 56) = 35.58, *p* < 0,0001; F (7, 104) = **(b)** 23.99, *p* < 0.0001; **(c)** 66.50, *p* < 0.0001; **(d)** 25.28, *p* < 0.0001. *p < 0.05, **p < 0.01, ***p < 0.001 vs respective age-CTR; °°°*p* < 0.001 vs respective age-MIA; $$*P* < 0.01, $$$*p* < 0.001 vs respective young adult treatment group

The development of anxiety after OA induction was also confirmed with the open field test, where a significant reduction in the distance traveled (panel d, *p* < 0.001) and of the time spent in the center zone of the arena (e, *p* < 0.001) was detected in both young adult and older adult MIA mice in comparison with age-matched CTRs. Moreover, we also registered the presence of compulsive-like behaviors in young adult and older adult MIA mice, as demonstrated by the lower number of marbles left unburied by these groups (panel g—CTR vs MIA young adult, *p* < 0.001; CTR vs MIA older adult, *p* < 0.05). All these anxiety-like responses in MIA mice were significantly ameliorated or normalized after 1-week treatment with morphine. As reported in Fig. 3, animals of the depression cohort were assessed in the sucrose preference (panel a), tail suspension (panel b) and forced swim (panels c-d) tests. In all tests, significant differences in the behavior were observed in CTR older adult mice in comparison with young adult ones. CTR older adult mice as reported in panel a, had a sucrose preference rate significantly lower than young adult CTRs (*p* < 0.001).

**Fig. 4:**
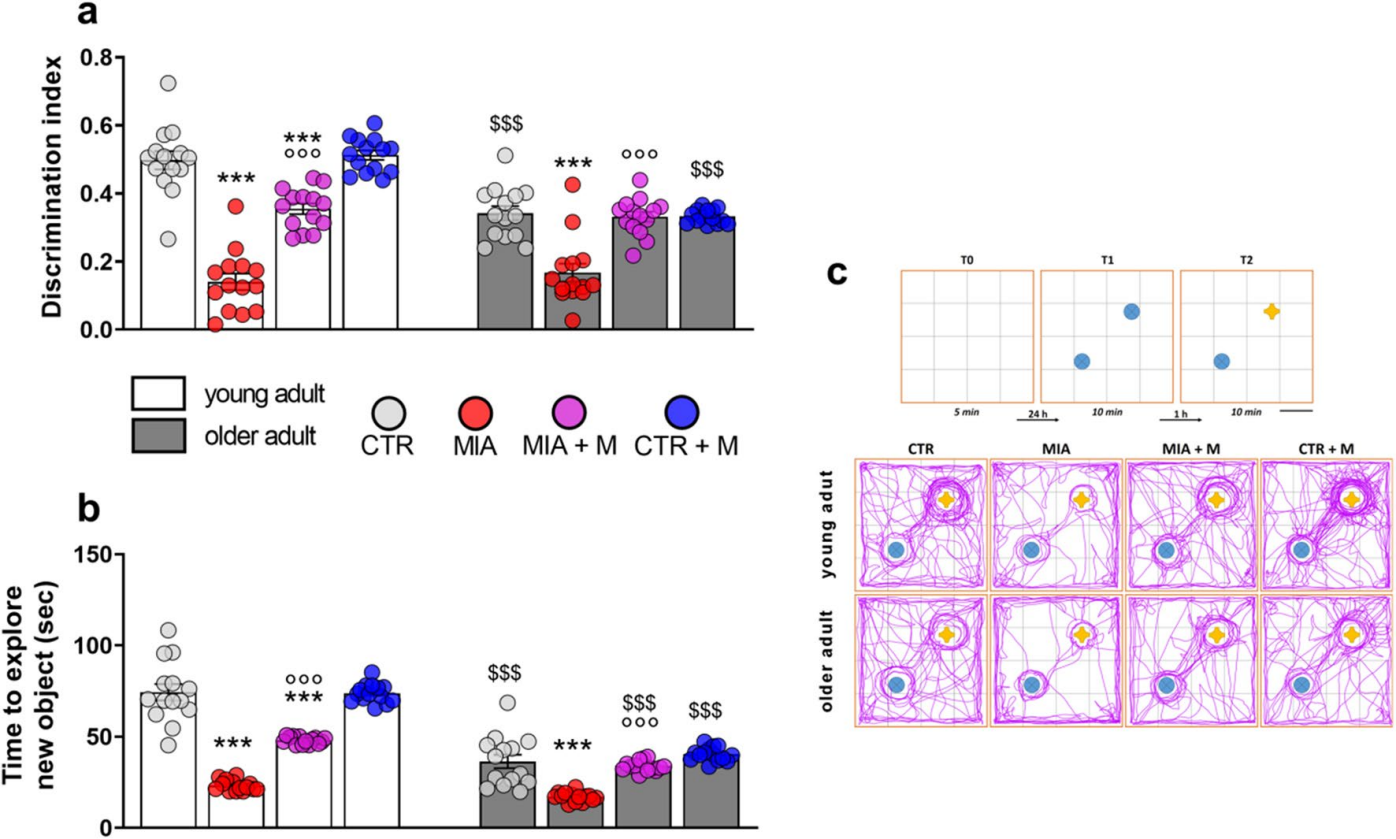
Cognitive deficit. Behavioral evaluations were performed at the end of the experimental protocol, at day 14 (MIA IA administered at day 0, 1 mg in 10 µl in the right knee; morphine, s.c. administered, 2.5 mg/kg, once daily, from day 7 until day 13). Cognitive impairment was assessed by the novel object recognition test, evaluating **(a)** the discrimination index and **(b)** the total time spent exploring the new object. **(c)** Schematic representation of trials and inter-trial intervals, and representative track plots of all experimental groups during the testing phase (T2). The familiar objects are represented in blue, while the new object is represented in yellow. Scale bar: 11.5 cm. Data are presented as mean ± SEM of 14 animals per group. Statistical analysis was performed by One-way ANOVA analysis of variance followed by Bonferroni’s post-test. Treatment: **(a)** F (7, 104) = 45.51, *p* < 0.0001; **(b)** F (7, 104) = 92.68, *p* < 0.0001. ***p < 0.001 vs respective age-CTR; °°°*p* < 0.001 vs respective age-MIA; $$$p < 0.001 vs respective young adult treatment group

At the TST, older adult animals had a significantly decreased mobility time (panel b, *p* < 0.001) and at the FST a reduction of the first immobility time (latency, panel c, *p* < 0.001) and an increase of the immobility time in the last 4 min of test (panel d, *p* < 0.001) was evident. In young adult mice, the presence of OA reduced sucrose preference (*p* < 0.001) and worsened responses in the TST and FST, suggesting a depressive-like behavior (TST, *p* < 0.001 and FST, *p* < 0.001), while it did not further aggravate the altered behavioral responses already present in older adult mice. In young adult animals, one week of morphine treatment was able to significantly prevent/ reduce the development of depressive behavior (*p* < 0.001). Moreover, we also observed that morphine administration in MIA older adult mice slightly ameliorated the behavioral responses in all the tests. However, morphine treatment did not affect any of the analyzed behavioral parameters in healthy young adult and older adult mice. Both aging and OA pain have been frequently associated with memory dysfunction in patients. Our results demonstrate that CTR older adult mice showed a decrease in both discrimination index (Fig. 4a) and exploration time towards the new object (Fig. 4b) compared to young adult CTRs (*p* < 0.001). Moreover, we found that MIA injection significantly impaired memory in young adult animals and further worsened it in older adult mice (*p* < 0.001). Morphine treatment was able to improve memory impairment in both young adult and older adult mice (*p* < 0.001).

### Biochemical evaluation

**Fig. 5:**
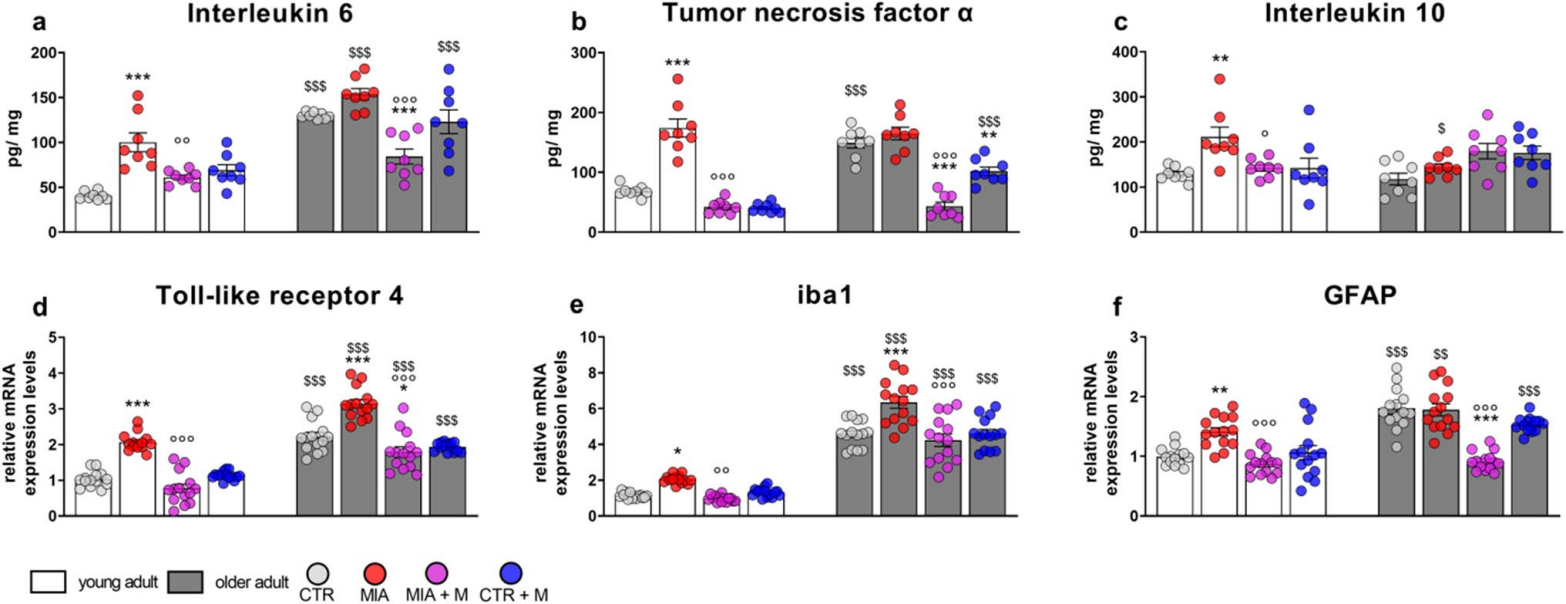
**| **Prefrontal cortex—PFC. Biochemical evaluations were performed at the end of the experimental protocol, at day 14 (MIA IA administered at day 0, 1 mg in 10 µl in the right knee; morphine, s.c. administered, 2.5 mg/kg, once daily, from day 7 until day 13). Protein levels of proinflammatory cytokines **(a)** IL-6 and **(b)** TNFα, and anti-inflammatory cytokine **(c)** IL-10 were measured by means of ELISA assay and reported as ratio between pg of cytokine and mg of total protein. The mRNA expression levels of **(d)** TLR4, **(e)** iba1 as microglia marker and **(f)** GFAP as astrocyte marker were assessed by means of Real Time-qPCR. Data were expressed in relation to GAPDH and presented as fold-changes over the levels of CTR young adult mice group. Data are expressed as the mean ± SEM from 8 (a and b) or 14 (c-e) mice per group. Statistical analysis was performed using One-way ANOVA followed by Bonferroni’s post-test. Treatment: F (7, 56) = **(a)** 26.64, *p* < 0.0001; **(b)** 42.34, *p* < 0.0001; **(c)** 4.348, *p* = 0.0007; F (7, 104) = **(d)** 66.69, p < 0.0001; **(e)** 98.45, *p* < 0.0001; **(f)** 26.91, p < 0.0001. *p < 0.05, **p < 0.01, ***p < 0.001 vs respective age-CTR; °*p* < 0.05, °°*p* < 0.01, °°°*p* < 0.001 vs respective age-MIA; $*p* < 0.05, $$*p* < 0.01, $$$*p* < 0.001 vs respective young adult treatment group

**Fig. 6:**
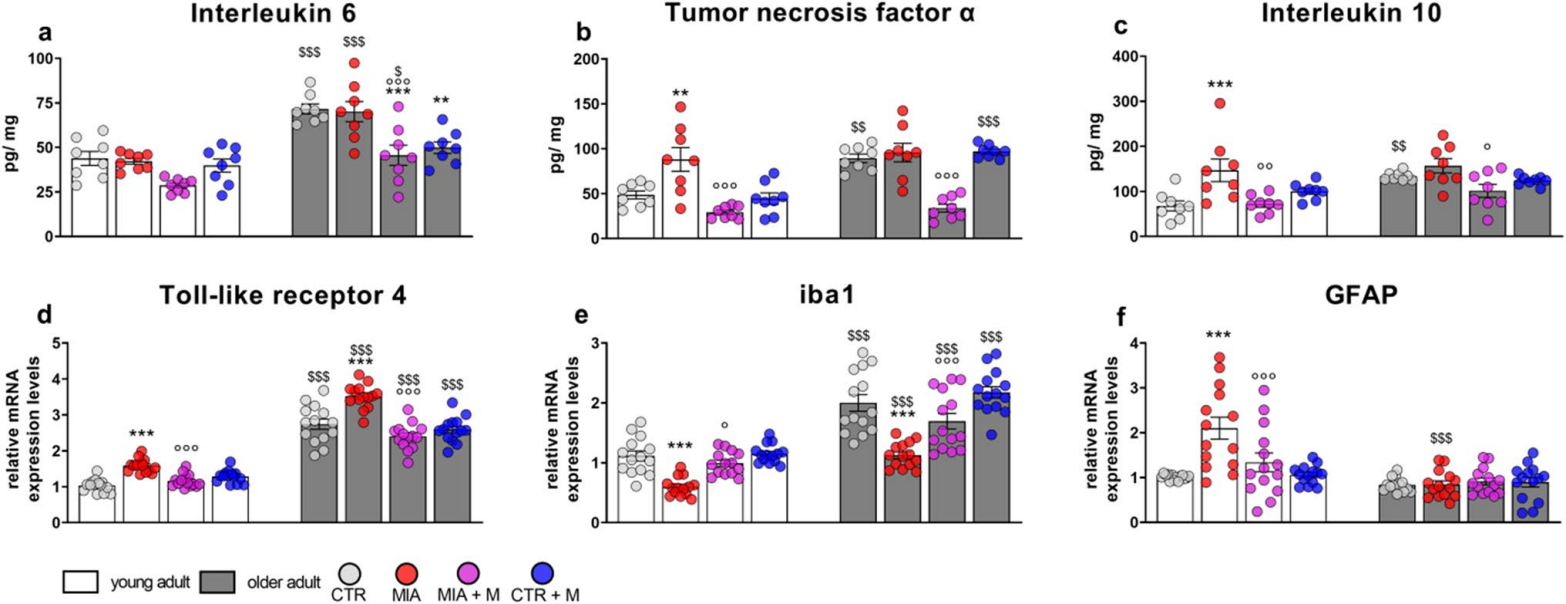
Hippocampus—HPC. Biochemical evaluations were performed at the end of the experimental protocol, at day 14 (MIA IA administered at day 0, 1 mg in 10 µl in the right knee; morphine, s.c. administered, 2.5 mg/kg, once daily, from day 7 until day 13). Protein levels of proinflammatory cytokines **(a)** IL-6 and **(b)** TNFα, and anti-inflammatory cytokine **(c)** IL-10 were measured by means of ELISA assay and reported as ratio between pg of cytokine and mg of total protein. The mRNA expression levels of **(d)** TLR4, **(e)** iba1 as microglia marker and **(f)** GFAP as astrocyte marker were assessed by means of Real Time-qPCR. Data were expressed in relation to GAPDH and presented as fold-changes over the CTR young adult mice group levels. Data are expressed as the mean ± SEM from 8 (a and b) or 14 (c-e) mice per group. Statistical analysis was performed using One-way ANOVA followed by Bonferroni’s post-test. Treatment: F (7, 56) = **(a)** 15.17, *p* < 0.0001; **(b)** 17.56, p < 0.0001; **(c)** 6.611, *p* < 0.0001; F (7, 104) = **(d)** 117.3, *p* < 0.0001; **(e)** 38.18, *p* < 0.0001; **(f)** 10.83, *p* < 0.0001. **p < 0.01, ***p < 0.001 vs respective age-CTR; °*p* < 0.05, °°*p* < 0.01, °°°*p* < 0.001 vs respective age-MIA; $*p* < 0.05, $$*p* < 0.01, $$$*p* < 0.001 vs respective young adult treatment group

**Fig. 7:**
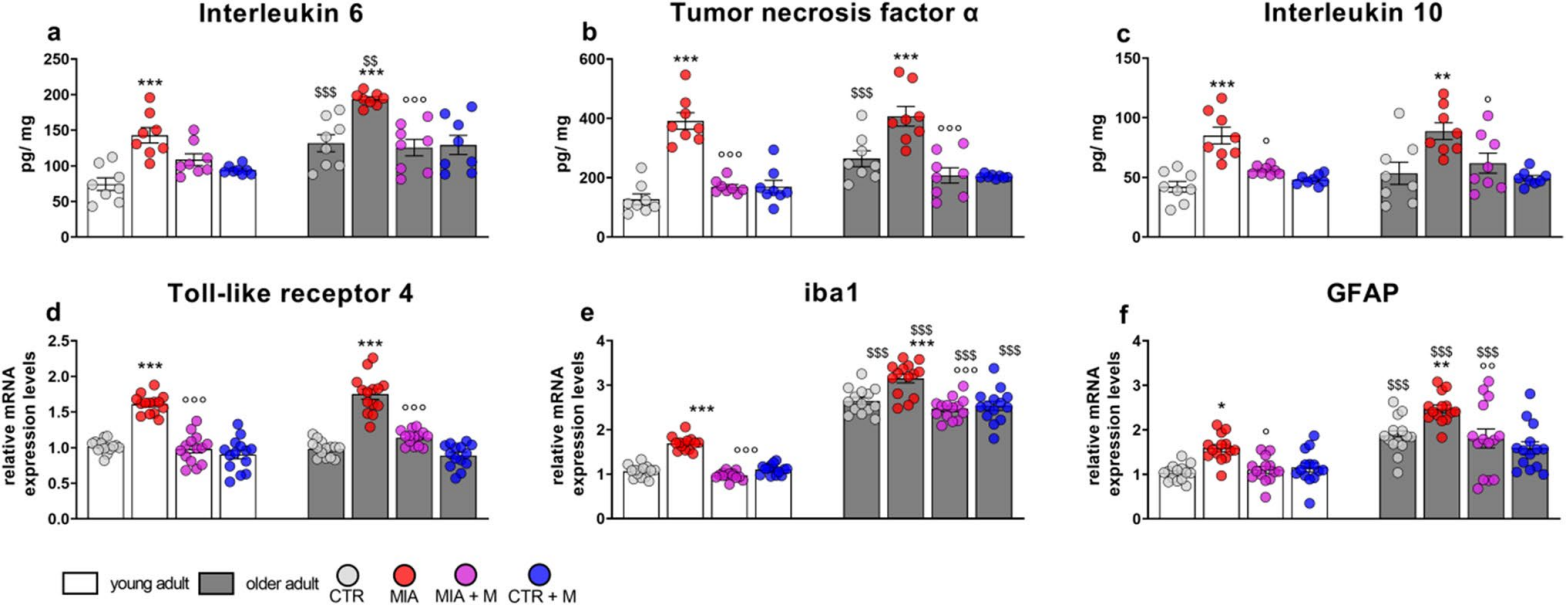
Hypothalamus—HPT. Biochemical evaluations were performed at the end of the experimental protocol, at day 14 (MIA IA administered at day 0, 1 mg in 10 µl in the right knee; morphine, s.c. administered, 2.5 mg/kg, once daily, from day 7 until day 13). Protein levels of proinflammatory cytokines **(a)** IL-6 and **(b)** TNFα, and anti-inflammatory cytokine **(c)** IL-10 were measured by means of ELISA assay and reported as ratio between pg of cytokine and mg of total protein. The mRNA expression levels of **(d)** TLR4, **(e)** iba1 as microglia marker and **(f)** GFAP as astrocyte marker were assessed by means of Real Time-qPCR. Data were expressed in relation to GAPDH and presented as fold-changes over CTR young adult mice group levels. Data are expressed as the mean ± SEM from 8 (a and b) or 14 (c-e) mice per group. Statistical analysis was performed using One-way ANOVA followed by Bonferroni’s post-test. Treatment: F (7, 56) = **(a)** 20.60, *p* < 0.0001; **(b)** 21.19, *p* < 0.0001; **(c)** 8.411, *p* < 0.0001; F (7, 104) = **(d)** 52.03, p < 0.0001; **(e)** 51.98, *p* < 0.0001; **(f)** 17.22, *p* < 0.0001. *p < 0.05, **p < 0.01, ***pp < 0.001 vs respective age-CTR; °*p* < 0.05, °°*p* < 0.01, °°°*p* < 0.001 vs respective age-MIA; $$*p* < 0.01, $$$*p* < 0.001 vs respective young adult treatment group

In prefrontal cortex (PFC, Fig. 5 and Supplementary Figure (SF) 2), hippocampus (HPC, Fig. 6 and SF2) and hypothalamus (HPT, Fig. 7 and SF2), proinflammatory cytokines IL-6 and TNFα (as protein – Fig. 5–7 and mRNA – Fig. SF2), the anti-inflammatory cytokine IL-10 (as protein – Fig. 5–7) and TLR4, iba1 (microglia) and GFAP (astrocytes) (as mRNA – Fig. 5–7), were evaluated. The levels of IL-6 (Fig. 5a and SF2) and TNFα (Fig. 5b and SF2) were higher in PFC of CTR older adult mice than those in CTR young adult mice (*p* < 0.001), indicating the presence of a basal neuroinflammatory status related to aging. However, IL-10 levels (Fig. 5c) were comparable between young adult and older adult CTRs. The presence of OA and chronic pain induced a significant cytokine increase in young adult mice (Fig. 5 – protein: IL-6 and TNFα, *p* < 0.001; IL-10, *p* < 0.01; SF2 – mRNA: IL-6, *p* = 0.1369; TNFα, *p* < 0.001) but did not further affect the cytokine levels in the older adult animals. One week of morphine treatment was able to prevent the IL-6 and TNFα rise in young adult animals (MIA vs MIA + M: IL-6, *p* < 0.01; TNFα, *p* < 0.001; IL-10, *p* < 0.05). Interestingly morphine administration was able to blunt the levels of the proinflammatory cytokines in both CTR and OA older adult mice.

In hippocampus IL-6 (Fig. 6a and SF2), TNFα (Fig. 6b and SF2) and IL-10 (Fig. 6c) levels were more elevated in older adult CTR animals in comparison with young adult ones (IL-6, *p* < 0.001; TNFα and IL-10, *p* < 0.01). In MIA older adult mice no further increase was observed, while in young adult MIA mice a significant upregulation of TNFα (*p* < 0.01) and IL-10 (*p* < 0.001) was evident. Morphine administration prevented TNFα and IL-10 overexpression in young adult MIA mice (*p* < 0.001 and *p* < 0.01, respectively) and reduced the age-related neuroinflammation observed in older adult mice (p < 0.05). Additionally, morphine treatment in CTR older adult mice significantly decreased IL-6 protein levels (CTR older adult vs CTR + M older adult, *p* < 0.01). In hypothalamus, as in the other two brain areas, IL-6 (Fig. 7a and SF2) and TNFα (Fig. 7b and SF2) levels were upregulated in CTR older adult mice in comparison with young adults (*p* < 0.001); while IL-10 (Fig. 7c) levels were not changed by aging. In this case, however, OA pain induced an increase in the levels of pro- and anti-inflammatory cytokines in both young adult and older adult MIA mice (Fig. 7 – protein: young adult—IL-6, TNFα and IL-10, *p* < 0.001; older adult—IL-6 and TNFα, *p* < 0.001; IL-10, *p* < 0.01; SF2 – mRNA: young adult—IL-6, *p* = 0.2131; TNFα, *p* < 0.001; older adult—IL-6 and TNFα, *p* < 0.001). Morphine treatment positively modulated pain-induced cytokine level increase in both young adult (MIA vs MIA + M: IL-6, *p* = 0.1744 (protein) and *p* = 0.2734 (mRNA); TNFα, *p* < 0.001; IL-10, *p* < 0.05) and older adult (IL-6 and TNFα, *p* < 0.001; IL-10, *p* < 0.05) MIA mice. In both young adult and older adult CTRs treated with morphine, no significant changes were observed in protein levels (CTR vs CTR + M, *p* > 0.05), while a significant decrease in the expression levels of the proinflammatory cytokines was observed in older adult CTRs treated with morphine (*p* < 0.05).

A neuroinflammatory condition in aging is further evidenced in CTR older adult mice that are characterized by higher mRNA levels of TLR4 in PFC (Fig. 5d, *p* < 0.001) and HPC (Fig. 6d, *p* < 0.001), of iba1 in PFC (Fig. 5e, *p* < 0.001), HPC (Fig. 6e, *p* < 0.001) and HPT (Fig. 7e, *p* < 0.001), and of GFAP in PFC (Fig. 5f, *p* < 0.001) and HPT (Fig. 7f, *p* < 0.001). In both age groups, OA condition caused a significant increase in TLR4 expression in all brain areas (p < 0.001). Moreover, in both young adult and older adult MIA mice an increase in iba1 levels was detected in the PFC (young adult, *p* < 0.05; older adult, *p* < 0.001) and HPT (*p* < 0.001), as well as an increase of GFAP in HPT (young adult, *p* < 0.05; older adult, *p* < 0.01). On the contrary, in MIA mice of both ages, a significant decrease of iba1 in HPC (*p* < 0.001) was present and only in MIA young adult mice was observed an upregulation of GFAP in PFC and HPC (PFC, *p* < 0.01; HPC, *p* < 0.001). In young adult and older adult MIA mice, chronic morphine treatment was able to decrease most neuroinflammatory markers and it also prevented the decrease of iba1 expression that was present in hippocampus, indicating that the treatment mitigated/ prevented the alterations observed in all three brain areas. The treatment did not alter any of the parameters analyzed in both young adult and older adult CTR mice.

**Fig. 8:**
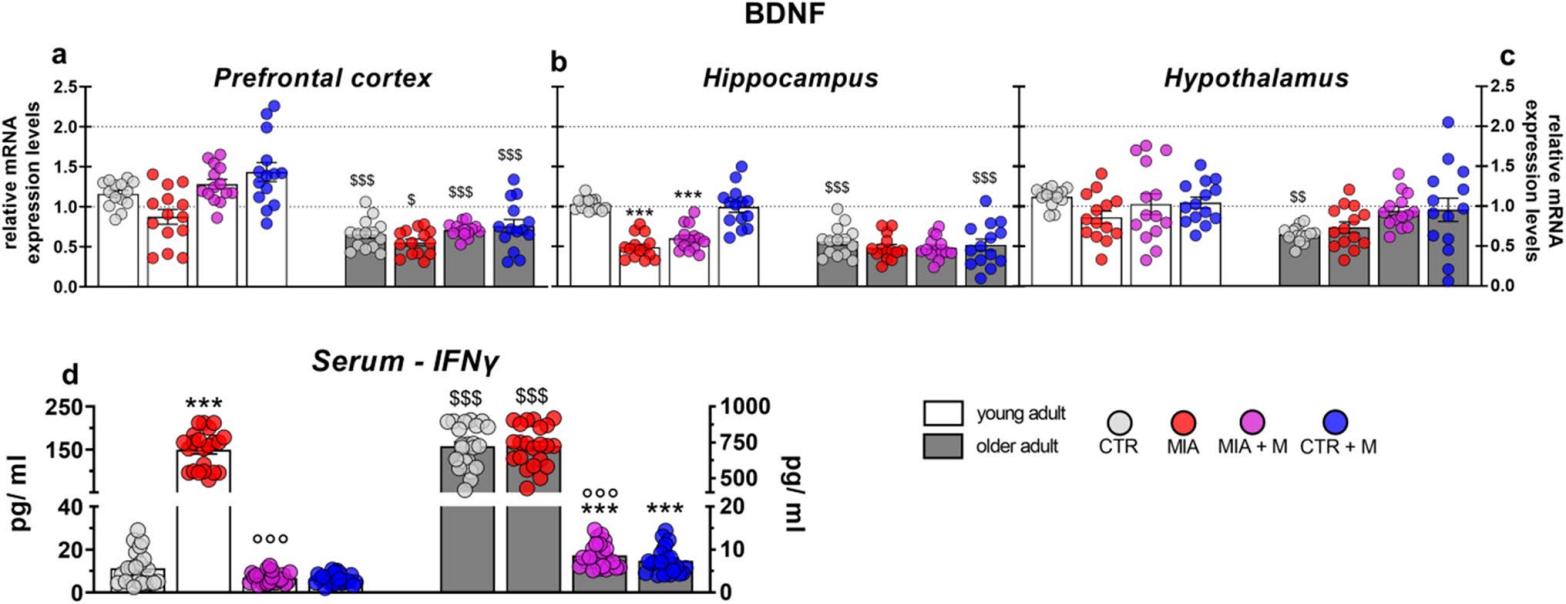
BDNF in brain areas and serum IFNγ levels. Biochemical evaluations were performed at the end of the experimental protocol, at day 14 (MIA IA administered at day 0, 1 mg in 10 µl in the right knee; morphine, s.c. administered, 2.5 mg/kg, once daily, from day 7 until day 13). The mRNA expression levels of BDNF were evaluated in **(a)** PFC, **(b)** HPC and **(c)** HPT by means of Real Time-qPCR. Data were expressed in relation to GAPDH and presented as fold-changes over the CTR young adult mice group levels. **(d)** Serum IFNγ levels were measured by means of ELISA assay. Data are expressed as the mean ± SEM from 14 (a-c) or 22 (d) mice per group. Statistical analysis was performed using One-way ANOVA followed by Bonferroni’s post-test. Treatment: F (7, 104) = **(a)** 16.05, *p* < 0.0001; **(b)** 22.24, *p* < 0.0001; **(c)** 3.51, *p* = 0.0020; **(d)** F (7, 168) = 418.2; *p* < 0.0001. ***p < 0.001 vs respective age-CTR; °°°*p* < 0.001 vs respective age-MIA; $*p* < 0.05, $$*p* < 0.01, $$$*p* < 0.001 vs respective young adult treatment group

In PFC (Fig. 8a), HPC (Fig. 8b) and HPT (Fig. 8c) the BDNF levels were lower in CTR older adult animals than in young adult ones (*p* < 0.01). In OA mice, a significant reduction of BDNF was observed only in HPC of young adult mice (*p* < 0.001), although a trend in this sense was also detectable in young adult MIA mice in the other two brain areas (PFC, *p* = 0.0575; HPT, *p* = 0.4211). Morphine treatment did not modify the neurotrophin level in any experimental group. Figure 8 also shows the serum levels of IFNγ, as the peripheral levels of this cytokine are frequently linked to a depressive state. IFNγ concentrations were significantly higher in serum from CTR older adult mice in comparison to young adult ones (*p* < 0.001) and MIA treatment did not affect them. In young adult animals, the presence of OA pain induced a significant increase in IFNγ levels (*p* < 0.001). The treatment with morphine was able to prevent the IFNγ increase in OA young adult mice (*p* < 0.001) but also to reduce it in both older adult CTR and MIA mice (*p* < 0.001).

The results of Pearson's correlation test showed a significant inverse correlation (Table 1, p < 0.001) between the decrease in BDNF mRNA levels and the increase of both IL-6 and TNFα proteins in PFC and HPC, while no correlation was observed in HPT. The same test also revealed a significant correlation (Table 2) between the increase of serum IFNγ levels and the immobility time in the last 4 min during the forced swim test (*p* < 0.0001). In addition, the peripheral increase of IFNγ was also inversely correlated with the decrease of BDNF in the brain areas (PFC, HPC and HPT).

**Table 1:** Tissue-specific correlation (PFC, HPC and HPT) between BDNF levels and proinflammatory cytokine levels

	PFC		HPC		HPT	
	IL-6	TNFα	IL-6	TNFα	IL-6	TNFα
BDNF	r =-0.5680	r =-0.4707	r =-0.2926	r =-0.2709	r =-0.1883	r =-0.1280
	*p* < *0.0001*	*p* < *0.0001*	*p* = *0.019*	*p* = *0.0304*	*p* = *0.1363*	*p* = *0.3155*

**Table 2:** Correlation between serum IFNγ levels and immobility time in FST and between IFNγ and BDNF levels in supraspinal areas

	FST	BDNF—PFC	BDNF—HPC	BDNF—HPT
IFN y	r = 0.4414	r =-0.4987	r =-0.2743	r =-0.3980
	*p* < *0.0001*	*p* < *0.0001*	*p* = *0.0034*	*p* < *0.0001*

## Discussion

In this study we suggest that the presence of chronic OA pain in young adult mice induces mood alterations and supraspinal biochemical modifications and aggravates behavioral abnormality and neuroinflammatory condition already present in older adult mice. Moreover, our results demonstrate that a prompt and efficacious treatment of chronic pain may prevent the development of anxio-depressive disorders and reduce neuroinflammation. Musculoskeletal pain, such as OA pain, is one of the main causes of disability related to pain, and OA patients are frequently affected by anxious and/ or depressive states as comorbidities (Rastogi and Meek 2013). In this study we used the MIA-induced OA model in young adult (11-week-old, approximately 20 years in humans) and older adult (20 months of age, approximately 60 years in humans) mice (Flurkey et al. 2007). MIA injection caused the development of a significant hypersensitivity that was similar in young adult and older adult animals, and one week of morphine treatment was able to contrast it. In this work, as a pain-like behavior parameter, we present only mechanical allodynia evaluated in the plantar paw, which is a generally accepted measure of referred hypersensitivity in MIA model (Ogbonna et al. 2015; de Sousa Valente 2019; Amodeo et al. 2021a; Amodeo et al. 2022). We are aware that these measurements, performed in the paw, may not completely reflect the pain phenotype that is present in affected joints of patients. However, in previous work with the same experimental MIA model and morphine treatment, in young adult and older adult mice (Amodeo et al. 2022), we performed a wider range of hypersensitivity assessments and we also obtained similar results in the weight bearing/incapacitance, hot plate and plantar tests. To date, the correlation between chronic pain and mood disorders has mainly been studied using preclinical animal models of neuropathic pain (Yalcin et al. 2011; Amodeo et al. 2021b), while less has been published with OA models (La Porta et al. 2015; Carcolé et al. 2019; Batallé et al. 2021), where both inflammatory and neuropathic pain components coexist (Thakur et al. 2014). These studies, however, were performed mainly with young adult animals, thus not considering aging as a variable. From the behavioral phenotyping here performed we found that older mice showed different parameters and responses from younger animals. In particular, in older adult mice, we observed cognitive impairment in the NOR test, reduced sucrose preference, and impaired yield behavior in TST and FST. In mice, the presence of these alterations is generally related to the presence of a depressive-like condition. We cannot, however, be sure that the observed modifications have a”psychiatric” significance, because they could also reflect aging alone. In OA young adult mice a chronic pain condition induced both a depressive-like state and a cognitive deficit, which displayed features resembling those observed in the CTR older adult mice. On the contrary, CTR older adult mice did not display an anxious phenotype compared to young adult ones, suggesting that aging itself does not affect this condition. However, it emerges from our data that the presence of chronic pain is a trigger for the development of anxiety-like behaviors both in young adult and older adult OA mice. In this respect, clinical and preclinical studies show that anxio-depressive symptoms are related to altered neuroplasticity in the cortico-limbic structures (Guida et al. 2022); it has also been demonstrated that both aging and chronic pain may lead to supraspinal alterations. In particular, on the one hand, during aging there is the presence of “neuroinflammAging”, i.e. low-grade inflammation that also affects the brain (Franceschi and Campisi 2014; D’Agnelli et al. 2022), and on the other hand, central sensitization that occurs during a chronic pain condition leads to the development of neuroinflammation in both spinal cord and brain (Ji et al. 2018). As confirmation of this, we have previously demonstrated that CTR older adult mice were characterized by significant neuroinflammation at sites involved in pain transmission and modulation (DRGs and spinal cord) compared to young adult ones (Amodeo et al. 2022). In particular, we observed that OA condition induced upregulation of proinflammatory cytokines and astrocytic and microglial activation in DRGs and spinal cord of young adult mice as well as severely exacerbated neuroinflammation already present in older adult animals (Amodeo et al. 2022). Here, we demonstrated that even at the supraspinal level in areas largely involved in pain perception and mood control such as hypothalamus (HPT), prefrontal cortex (PFC) and hippocampus (HPC) the levels of proinflammatory cytokines IL-6 and TNFα and the expression levels of TLR4 and of the microglia marker iba-1 were significantly higher in CTRs older adult than in young adults. In the same mice it was also evident an increase of IL-10 in HPC. These results agree with other studies that reported, in both rodent and human brain, that markers generally related to the activated microglia such as iba1, CD11b and TLRs are elevated in aged microglia together with higher levels of proinflammatory cytokines (Finch et al. 2002). As microglia, astrocytes are also affected by physiological aging. In particular, it has been demonstrated that in the aged brain astrocytes are hypertrophic with consequent alteration of intracytoplasmic antigens, such as GFAP (Cotrina and Nedergaard 2001). In accordance with this, our data show higher GFAP levels in CTR older adult hypothalamus and prefrontal cortex, but not in the hippocampus. Furthermore, in CTR older adult mice compared to young adults, we also detected a BDNF downregulation in all the brain areas considered. It is well known that lower BDNF levels are associated with a depressive state (Lee and Kim 2010). Moreover, recent evidence indicated that depression is related to serum upregulation of proinflammatory cytokines, including IFNγ (Chen et al. 2021). In our study we observed that older adult CTR mice showed significantly higher IFNγ levels than young adult CTRs. Therefore, the presence of neuroinflammatory conditions associated with low BDNF levels in the supraspinal areas and high IFNγ serum levels, together with the altered behavioral responses in TST and FST may support the concept of a depressive-like state in CTR older adult animals. We observed that the impact of chronic pain on neuroinflammation is different in young adult and older adult mice. In general, the presence of pain induced a significant upregulation of most proinflammatory cytokines and an alteration of glial activation markers in adult brain areas, while the aged brain, already characterized by neuroinflammation, was less altered. In HPC we found an inverse correlation between proinflammatory cytokines and BDNF levels in PFC and HPC. Additionally, the presence of chronic pain in older adult OA mice further increases all the neuroinflammatory parameters at the hypothalamic level. The activation of anti-inflammatory cytokine IL-10 is an important signal counterbalancing neuroinflammation. It is interesting the fact that while the presence of pain in young adult animals is accompanied by a significant increase in IL-10, this anti-inflammatory signal is scarcely modulated in older adult animals, suggesting the higher vulnerability of the aged brain to stressful insults. We could therefore speculate that CTR young adult mice, starting from a healthy basal condition, were more prompt to react to the presence of pain, while the older adult mice, characterized by an inflammatory condition already present, may react to the pain stress with different timing. It is indeed possible that longer exposure to pain may induce a more evident modulation also in aged mice. Furthermore, it is interesting to note that in HPC of both young adult and older adult OA mice we surprisingly found a significant reduction in iba1 expression levels. Although in contrast to what has been observed for other areas, a similar reduction of activated microglia has also been reported in other painful conditions (Kreisel et al. 2014; Amodeo et al. 2021b) where it has been shown that prolonged microglia activation may lead to apoptosis or a dystrophic phenotype of these cells, suggesting that stressed factors/ stimuli could lead to the establishment of dynamic neuroplasticity. However, a comprehensive explanation for this microglial downregulation in young adult and older adult mice when exposed to pain is currently lacking. We know that an immunofluorescence analysis would have helped to correlate the biochemical modifications observed in the brain areas to glial morphological changes and identify the cells mainly responsible for the alteration in aging and OA pain. This lack, therefore, represents a limitation of our paper. However, the presence of brain neuroinflammation clearly emerges from the panel of cytokines and glial markers which we analyzed as proteins and/or mRNAs. An important result from our study is the positive effect exerted by 1-week of morphine treatment on both behavioral responses and brain areas’ alterations in young adult and older adult OA mice. Indeed, to date, efficacious treatment of chronic musculoskeletal pain in the elderly is still a relevant problem (El-Tallawy et al. 2021). It is well known that, although efficacious, opioid treatment is accompanied by several unwanted effects that may limit its use (Pergolizzi et al. 2008; Rastogi and Meek 2013). However, in MIA model we and others demonstrated that morphine treatment significantly relieves chronic pain both in young adult and older adult mice (Han et al. 2021; Amodeo et al. 2022). Indeed, morphine prevented in OA mice, the development of anxiety-like symptoms, and blocked the development of depression-like behavior in young adult mice suggesting that the effect is due to the important pain relief induced by the opioid. This improvement in anxio-depressive state in mice seems to be well reflected by the modulation exerted by morphine on neuroinflammation in brain areas. The interplay among morphine, opioid receptors and neuroinflammation is complex and multifaceted (Cuitavi et al. 2022). This aspect has been extensively studied in the last years, with a particular focus, however, on spinal cord and less on supraspinal areas. The impact exerted by morphine on neuroinflammation is debated, with data from the literature that are often contradictory, showing both an increase (Cahill and Taylor 2017; Reiss et al. 2022) and a decrease (Chao et al. 1997; Zhang et al. 2020) in central neuroinflammation. Some of these studies seem to indicate that morphine itself may activate microglia, while others suggest that the opioid exerts significant generalized immunosuppression (Franchi et al. 2019; sacerdote et al. 2012).

Our data clearly indicate that one week of morphine treatment, at a dose able to relieve pain, was able to blunt the activation of microglia, astrocytes and overexpression of proinflammatory cytokines induced by both aging and pain. However, it must be considered that it is possible that the dose and the duration of the morphine treatment may be critical in order to either blunt or trigger microglia activation (Carcolé et al. 2019). We are aware that one limitation of our study is that all evaluations were only performed up to 2 weeks after OA induction. Indeed, it cannot be excluded that a longer treatment period may elicit different results, but the aim of this study was to unveil the impact of prompt pain treatment on the development of depression and anxiety, comorbidities that often accompany chronic pain patients. We must underlie that our data also suggest that the duration of chronic pain is important for the induction of anxiety- and/ or depressive-like behaviors. Indeed, while significant mood alterations are observed 2 weeks after OA induction in untreated mice, in morphine-treated animals in which pain was present for only 1 week, the anxio-depressive state was comparable to that of CTR mice, further indicating the importance of precocious pain treatment in order to modify the development of co-morbidities. Furthermore, we are also aware that the length and the dosage of the treatment used in our study cannot be fully comparable to the duration of chronic pain treatment that produces adverse effects in patients. Moreover, considering the well-known problems related to the chronic use of opioids, such as the potential development of addiction, that significantly negatively impacted general health in the last years, we are aware that other novel approaches that may relieve pain should be actively searched (Humphreys et al. 2022). Additionally, it is interesting to underline that our analysis revealed the presence of a direct correlation of serum IFNγ with depressive-like behavior (FST) and of a negative correlation with central BDNF levels in all animals. These observations led us to speculate about the fascinating hypothesis to use circulating IFNγ as a marker of glia alterations that can also help to monitor clinical response to analgesic treatment (Garden and Campbell 2016).

In conclusion, since the presence of chronic pain is characterized by a neuroinflammatory condition in specific brain areas involved in mood and cognitive control that resemble what we observed in older adult mice, we would like to hypothesize that pain and aging could share some common mechanisms and pathways (D’Agnelli et al. 2022). Although in our experimental setting morphine treatment is beneficial to treat OA pain, modulate related mood disorders such as depression and anxiety, and downregulate supraspinal neuroinflammation, caution is needed to translate these results to patients.

## Supplementary Information

Below is the link to the electronic supplementary material.

Supplementary file1 (DOCX 3800 KB)

## Data Availability

Data will be made available by authors upon reasonable request.
