# Supplementary material for: Supraspinal neuroinflammation and anxio-depressive-like behaviors in young- and older- adult mice with osteoarthritis pain: the effect of morphine
Source: Psychopharmacology (Berl). 2023 Aug 2;240(10):2131–46. doi: 10.1007/s00213-023-06436-1 (PMC10506934; doi:10.1007/s00213-023-06436-1)

**Supplementary Figure 1 Schematic experimental protocol.**

**
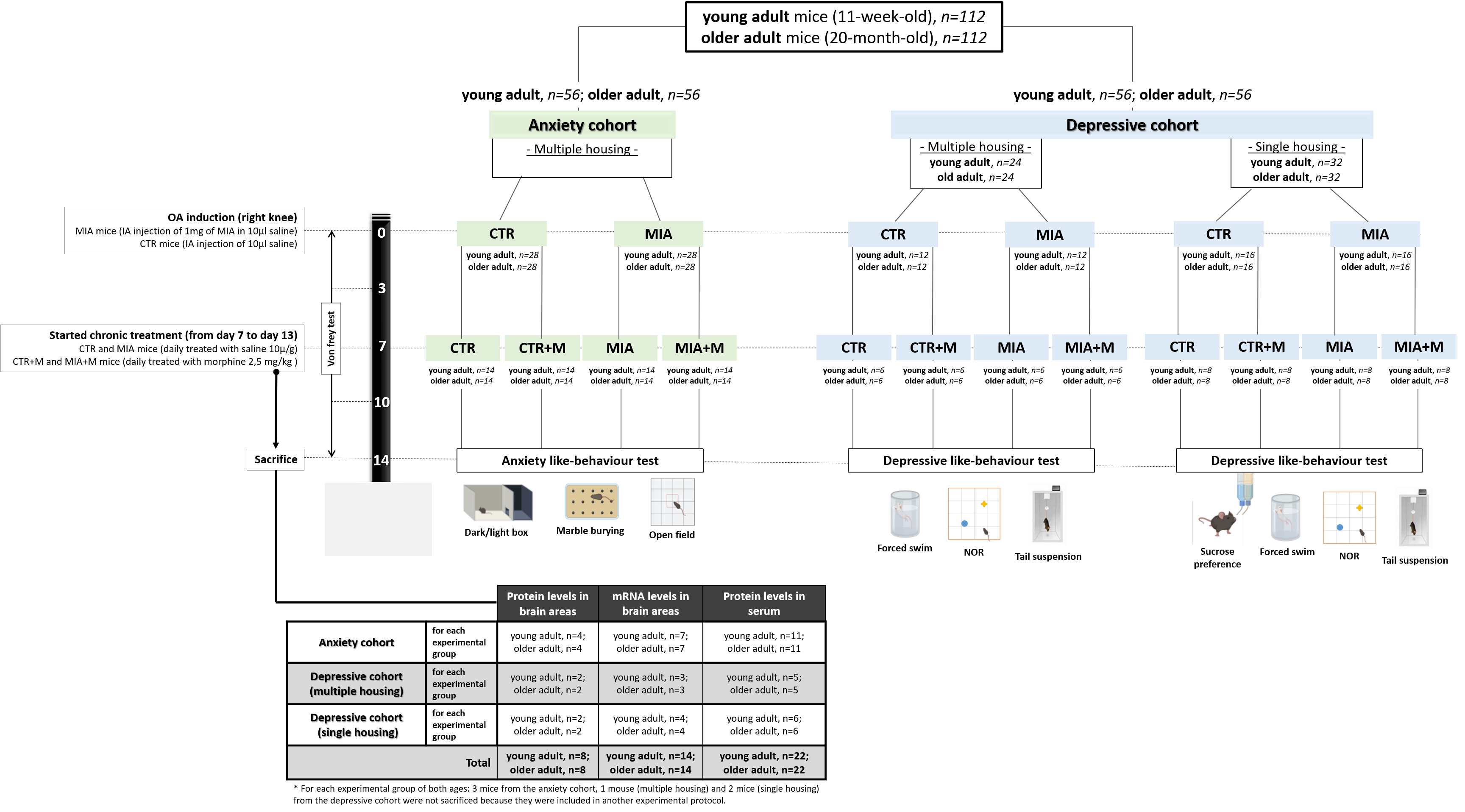
**

**Supplementary Figure 2 Expression levels of proinflammatory cytokines in brain areas.** Biochemical evaluations were performed at the end of the experimental protocol, at day 14 (IA MIA administered at day 0, 1mg in 10µl in the right knee; morphine, s.c. administered, 2.5 mg/kg, once daily, from day 7 until day 13). The mRNA expression levels of **(A)** IL-6 and **(B)** TNFα were evaluated in PFC, HPC and HPT by means of Real Time-qPCR. Data were expressed in relation to GAPDH and presented as fold-changes over the levels of CTR young adult mice group. Data are expressed as the mean ± SEM from 14 mice per group. Statistical analysis was performed using One-way ANOVA followed by Bonferroni’s post-test. Treatment: F (7, 104) = **(A)** PFC, 13.59, p<0.0001; HPC, 22.79, p<0.0001; HPT, 35.61, p<0.0001; **(B)** PFC, 33.19, p<0.0001; HPC, 84.61, p<0.0001; HPT, 87.82, p<0.0001; *p<0.05, **p<0.01, ***p <0.001 vs respective age-CTR; °°°p<0.001 vs respective age-MIA; $p<0.05, $$p<0.01, $$$p<0.001 vs respective young adult treatment group.


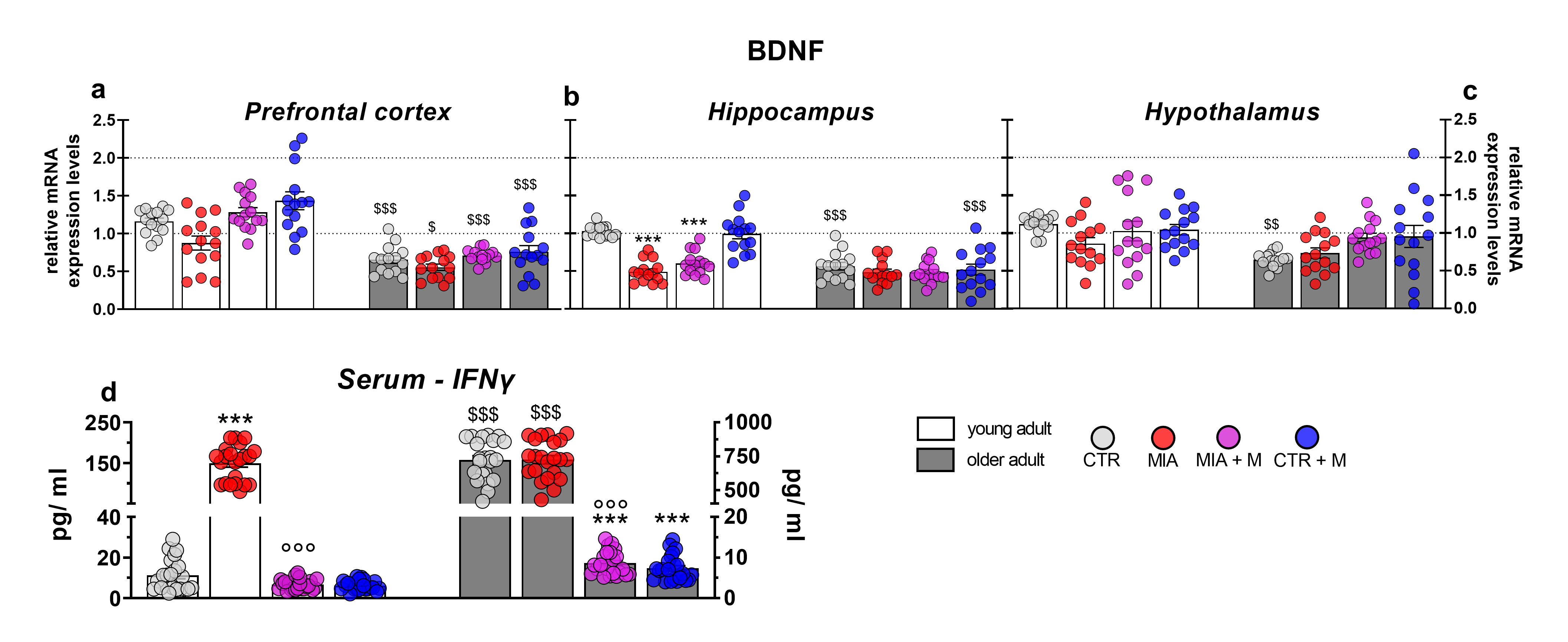

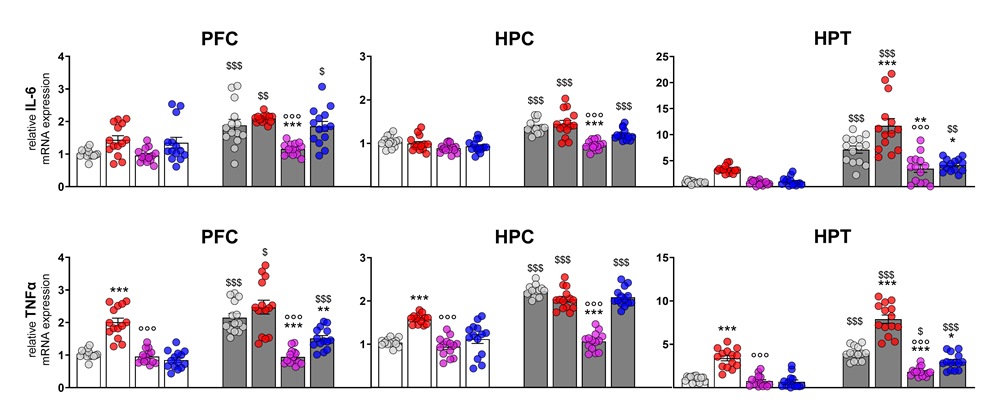

Supplement: Supplementary file 1 — Supplementary file1 (DOCX 3800 KB) [file 213_2023_6436_MOESM1_ESM.docx]
